# Treatment of Spinal Epidural Abscess With Limited Decompression

**DOI:** 10.31486/toj.24.0132

**Published:** 2025

**Authors:** Alhasan Alani, Spencer Taylor, Charles Yu

**Affiliations:** ^1^ The University of Queensland Medical School, Ochsner Clinical School, New Orleans, LA; ^2^ Department of Orthopedic Surgery, Ochsner Clinic Foundation, New Orleans, LA

**Keywords:** *Decompression*, *epidural abscess*, *laminectomy*, *surgical procedures–operative*

## Abstract

**Background:**

Spinal epidural abscess, the collection of purulent material in the epidural space, can cause spinal cord compression and neurologic deficits, including paralysis. Management of spinal epidural abscess includes antibiotic therapy and surgical decompression. Limited evidence is available to guide the extent of surgical decompression necessary for symptom and abscess resolution. Treatment depends on a variety of factors including the size, orientation (ventral or dorsal), and location of the spinal epidural abscess, along with patient-specific factors such as neurologic status.

**Case Report:**

A 75-year-old male presented with increasing back pain and altered mental status 7 weeks after a motor vehicle crash. The patient had elevated inflammatory markers and was without focal neurologic deficit. Advanced imaging demonstrated a ventral abscess from C2 to T11, a dorsal abscess extending from T9 to T12, ventral and dorsal abscesses from L1 to the sacrum, and myelomalacia from T10 to T12. Limited decompression was performed in the form of T9 to L1 laminectomy for evacuation of the abscess, followed by irrigation with a pediatric Foley catheter. Imaging obtained after surgical intervention and intravenous antibiotic therapy demonstrated complete resolution of the abscesses. The patient's symptoms resolved, and he was doing well at postoperative follow-up.

**Conclusion:**

Our case suggests that extensive decompression may not be necessary for treatment of a large spinal epidural abscess, thus preserving the structural integrity of the spine and potentially minimizing morbidity.

## INTRODUCTION

Spinal epidural abscess is the collection of purulent material in the epidural space and can cause spinal cord compression and neurologic deficits.^1^ Symptoms progress in a series of 4 stages. The first stage of presentation is back pain, followed by nerve root pain from the involved level, then neurologic changes (such as weakness, sensory loss, and bowel and bladder changes), and finally paralysis.^2-5^ A classic triad of fever, back pain, and neurologic deficit has previously been described, although presentation with the triad is not common.^3,5-8^ For example, in a series of 101 patients with spinal epidural abscess, only 8% presented with the triad.^9^

In addition to fever, back pain, and neurologic deficit or injury, laboratory evaluation commonly shows elevations in white blood cell count, erythrocyte sedimentation rate, and C-reactive protein.^3,5-8,10-12^ Spinal epidural abscess is often misdiagnosed as other medical conditions, including both infectious and noninfectious etiologies, which may delay surgical treatment and lead to progression of injury and neurologic deficit.^3-8^ As such, imaging modalities such as magnetic resonance imaging (MRI) and computed tomography scan can aid in diagnosis and direct treatment.^3,5,10,13,14^ MRI is highly sensitive, able to detect infections in as early as 1 week, and able to show the extent of the infection for surgical planning.^12^

In 1992, Darouiche et al reported an incidence of spinal epidural abscess of 0.29 to 0.80 per 10,000 hospital admissions, with a trend toward an increase in the decades studied.^4^ This trend of increasing incidence continues in part because of an increase in risk factors, including diabetes mellitus, intravenous drug use, end-stage renal disease, trauma, HIV infection, and prior surgical or procedural intervention to the spine.^3-5,9-11,13^ In addition, older age (>60 years), diabetes mellitus, respiratory and renal conditions, disseminated cancer, and thrombocytopenia are associated with 30-day mortality following surgical intervention for spinal epidural abscess.^15^ The predictive algorithm developed by Du et al demonstrated that patients with 4 or more of the listed risk factors had a 30-day mortality rate of 37.5% compared to the 30-day mortality rate of 3.7% among the full population of patients who underwent surgery for spinal epidural abscess.^15^

The mechanism of pathogenesis is either contiguous spread or, more commonly, hematogenous dissemination, but in many cases the cause of the infection is not identified.^3^ The most common pathogen to cause spinal epidural abscess is *Staphylococcus aureus*, with an increasing trend toward infection by methicillin-resistant *S aureus* (MRSA).^3,4,7-9,13,16^ Patients with MRSA infection have been shown to have a worse prognosis and increased failure of nonoperative treatment compared to patients infected with other pathogens.^13,16,17^ In a study of 101 patients with spinal epidural abscess, the 7 patients who died were infected with *S aureus*.^9^

Management of spinal epidural abscess begins with empiric antibiotic treatment that is then limited to directed antibiotic treatment once the pathogen is identified.^5^ Whether surgical management should also be included in the initial treatment phase is debated. In some settings, nonoperative treatment may be appropriate, such as in patients without neurologic injury, those whose overall medical condition contraindicates surgery, those with panspinal involvement of the abscess, and those with paralysis for an extended period.^3,5,13^ Nonoperative treatment of spinal epidural abscess has increased, and the increase in medical management may be attributable to earlier detection of spinal epidural abscess through sensitive imaging modalities such as MRI.^17^ Karikari et al found that nonoperative treatment was more likely to be successful in patients with ventral abscesses vs dorsal abscesses.^18^

Surgical indications for spinal epidural abscess include neurologic injury, spinal instability, and continued infection despite appropriate antibiotic therapy.^16^ Surgical management commonly includes decompressive laminectomy,^5,9^ but laminectomy alone may not be sufficient, as infections that involve multiple levels may need more extensive decompression and thus necessitate the need for fusion to stabilize the spine.^5^ Surgical technique is also dependent on the location of the abscess; both anterior and posterior approaches are used.^5^ In a case series of 2 patients with holospinal epidural abscess (an abscess that forms in the entirety of the spinal canal), 1 patient required surgical intervention using both an anterior and posterior approach and the other patient required segmental laminectomies.^19^

Overall, treatment of spinal epidural abscess does not have a clear consensus and depends on a variety of factors related to the infection, surgical approach, and patient. Further, spinal epidural abscess is a rare and serious condition, making it challenging to conduct large-scale or randomized trials to help establish guidelines. Ultimately, management goals include abscess drainage, spinal cord decompression, spinal stabilization, early mobilization, and neurologic recovery.

We present a case of a spinal epidural abscess that was treated with limited decompression via a T9 to L1 laminectomy, with complete resolution of the abscess at 2-month follow-up.

## CASE REPORT

A 75-year-old male with a history of hemorrhagic stroke, diabetes mellitus, stage 3 chronic kidney disease, chronic proximal left lower extremity deep vein thrombosis, and hypertension presented to the emergency department (ED) with a 1-week history of worsening back pain and altered mental status. The patient had been involved in a motor vehicle crash 7 weeks prior. He reported continued back pain for 1 month that was unrelieved by oral pain medications. The patient had visited an outside orthopedic surgeon who prescribed steroids and narcotic pain medication that caused short-term amnesia, confusion, worsening expressive aphasia, and 2 falls without head trauma. The patient was taken to the Veterans Affairs (VA) hospital ED after he was found on the bathroom floor and was transferred to our facility when an MRI revealed extensive epidural fluid collection.

The patient's surgical history included previous spinal surgery; however, the date and procedure are unknown. Upon presentation, he had back pain and altered mental status but no other neurologic symptoms. Physical examination demonstrated 5/5 strength, 2+ reflexes, and normal sensation in upper and lower extremities. The patient also had oral decay and a lower back burn wound from a heating pad.

**Figure 1.**
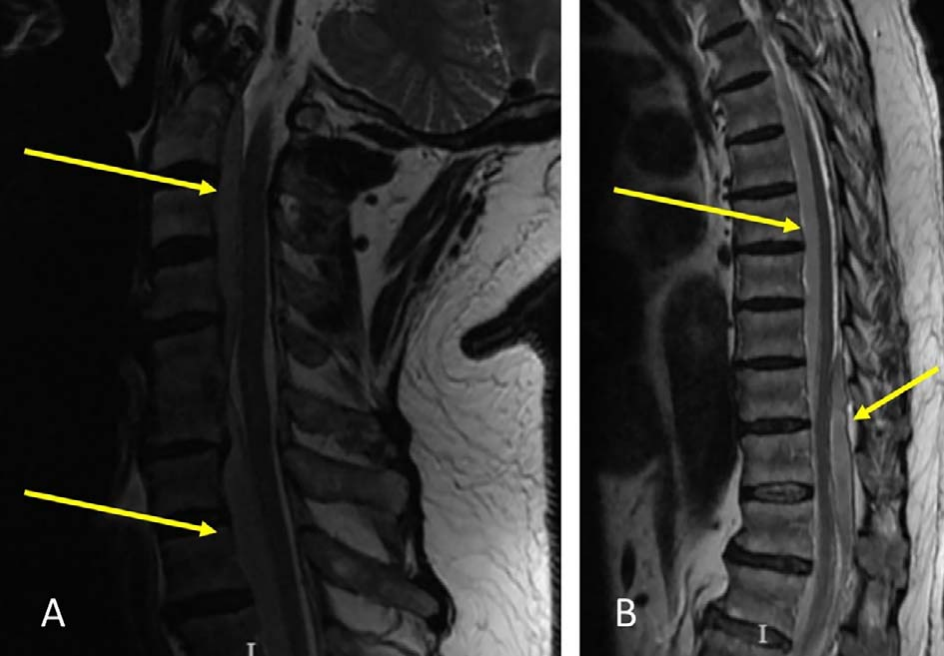
Preoperative sagittal T2 magnetic resonance imaging sequence of (A) the cervical spine and (B) the thoracolumbar spine shows epidural abscess formation (arrows).

Spine MRI obtained at the VA ED demonstrated a ventral abscess from C2 to T11, a dorsal abscess extending from T9 to T12, ventral and dorsal abscesses from L1 to the sacrum, and myelomalacia from T10 to T12 (Figure 1). Laboratory workup revealed elevated erythrocyte sedimentation rate of 80 mm/h (reference range, 0-10 mm/h), C-reactive protein of 457 mg/L (reference range, 0.0-8.2 mg/L), and white blood cell count of 14.7 K/μL (reference range, 3.90-12.70 K/μL). Blood cultures were taken in the ED.

Given the patient's presentation and MRI findings, a T9 to L1 laminectomy for abscess evacuation was undertaken after his international normalized ratio was increased to 1.1 from 1.0 at admission.

Prior to surgical incision, standard equipment was placed for neuromonitoring of motor evoked potentials (MEPs) and somatosensory evoked potentials (SSEPs). Because of the patient's deep vein thrombosis history and the need to cease anticoagulation medication for an extended period, the vascular surgery team placed an inferior vena cava filter prior to surgery. The surgery began immediately after the filter placement.

First, T9, T10, T11, and T12 laminectomies were performed to decompress the spinal cord and nerve roots. A large amount of white cloudy purulence was visualized and cultured. The thecal sac was decompressed using a curette and Woodson elevator. After initial decompression and debridement, additional purulence was encountered caudal to the site of the T12 laminectomy. Because of the extent of the additional purulence, an additional laminectomy of L1 was performed to allow for more thorough irrigation and decompression.

A pediatric Foley catheter was passed dorsal to the thecal sac and distal to the L2 lamina into the spinal canal. A total of 7 cm of catheter was passed through the canal to break up purulence, gently aspirate and irrigate the spinal canal, and debride additional purulence. The catheter was passed through the canal multiple times until no purulence was visible. The wound was then irrigated with sterile saline and dilute Betadine solution. MEPs and SSEPs were consistent with baseline and showed no evidence of neurologic injury at the completion of the procedure.

Three grams of vancomycin powder was placed in the deep space, and a drain was placed and set to gravity. The deep fascia was closed. Following closure of the deep fascia, a superficial drain was placed prior to skin closure and set to suction. The superficial layer was irrigated and closed over an additional 1 g of vancomycin.

The patient was allowed weight-bearing as tolerated with spine precautions via the use of a lumbar sacral orthosis brace.

The patient was treated empirically with 2 g of intravenous (IV) ceftriaxone every 12 hours and 1,500 mg of IV vancomycin every 12 hours following surgical intervention. Blood cultures from admission and surgical cultures grew *Streptococcus intermedius*. On postoperative day (POD) 3, Infectious Disease recommended discontinuing the vancomycin and administering 2 g of IV ceftriaxone every 12 hours for 6 weeks. On POD 10, blood cultures from POD 1 again grew *S intermedius*, so the patient was placed back on 1,500 mg of IV vancomycin every 12 hours because of the risk of resistant *S intermedius*. On POD 12, vancomycin was discontinued after repeat blood cultures showed no growth. On POD 13, the final Infectious Disease recommendation was 2 g of IV ceftriaxone for 8 weeks.

During his hospitalization, the patient had waxing and waning encephalopathy that gradually improved with supportive care. He was discharged on POD 19 (21 days from admission) to a rehabilitation center.

Five days after discharge, the patient presented to the ED with abdominal pain. He was found to have a multicystic abscess on his right psoas muscle. Per Infectious Disease recommendation, the patient's antibiotic therapy was changed to 1,050 mg of IV daptomycin every 24 hours because of concern for resistance. Interventional Radiology aspirated the abscess and placed a drain. Although the abscess had decreased in size 8 days later, it had developed a loculated appearance, so another drain was placed.

**Figure 2.**
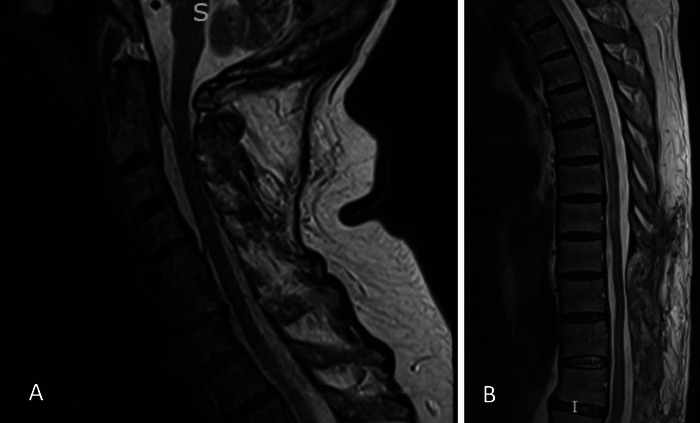
Postoperative sagittal T2 magnetic resonance imaging sequence of (A) the cervical spine and (B) the thoracolumbar spine shows resolution of epidural abscess.

At follow-up 8 weeks after the T9 to L1 laminectomy and epidural abscess evacuation, the patient was doing well overall. His pain had resolved, he reported subjective improvement in strength, and he was ambulating with a cane. MRI obtained at the follow-up appointment showed complete resolution of the epidural abscess throughout the spine (Figure 2), except for mild residual fluid collection by the sacrum. The patient was allowed to discontinue use of the lumbar sacral orthosis brace as tolerated. At 4-month follow-up, thoracolumbar x-ray showed chronic degenerative changes in addition to postoperative changes. The patient continued to do well without any recurrence of spinal epidural abscess.

## DISCUSSION

Spinal epidural abscess is a rare condition, with variability among patients in terms of anatomic location, orientation, size, and risk factors. Because of this rarity and variability, the decision to use medical or surgical management is a challenging one, as is the choice of surgical treatment option. For example, in a retrospective review by Chaker et al, the authors found a higher rate of reoperation rates and need for postoperative transfusion in patients undergoing both laminectomy and fusion compared to fusion alone.^20^ And although the treatment cost for laminectomy alone is lower than for laminectomy and fusion, laminectomy alone has a higher risk of spinal instability that increases with additional levels of surgery.^20-22^ A benefit of spinal fusion is spinal stability, and for treating certain conditions, such as lumbar spondylolisthesis, improved outcomes have been reported for patients undergoing laminectomy and fusion vs laminectomy alone.^21,23^ Another consideration is that spinal fusion frequently involves the use of spinal instrumentation, and instrumentation use in the setting of infection has a theoretical risk of increased infection recurrence. The connection between instrumentation and increased infection recurrence has not been definitively shown, however, and studies have shown the safety of instrumentation in the setting of infection.^24,25^

There is also uncertainty regarding the extent of laminectomy for treatment of spinal epidural abscess. Bridges and Than found that 3 of 4 patients with spinal epidural abscess who underwent focal laminectomy at the site of mass effect required a return to the operating room with deteriorating status.^26^ In the Smith et al review of 19 cases of holospinal epidural abscess, patients underwent a variety of treatments, including nonoperative treatment and multiple levels of laminectomies.^27^ In the Lau et al case series, a patient with holospinal epidural abscess was treated with segmental laminectomies and catheter irrigation, similar to our patient.^19^ Lau et al pointed out that treatment of holospinal epidural abscess usually involves either segmental laminectomies with catheter irrigation or radical laminectomies of the spine, but radical laminectomies pose the risk of postlaminectomy kyphosis because of the decreased stability of the spine.^19^

The differences in outcomes and treatment options for spinal epidural abscess warrant further research, but because of the rarity and severity of the condition, targeted studies are highly unlikely. In 2006, Darouiche proposed a treatment algorithm for medical and surgical management based on the extent of infection, operative risk, and neurologic injury.^3^ In 2018, Shah et al developed nomograms based on independent risk factors for both pretreatment motor deficit and 90-day mortality to aid in decision-making.^1^

The literature provides little to no guidance on specific surgical interventions, likely because of variable patient- and infection-related factors. The future study of spinal epidural abscess will likely continue to rely on retrospective analyses, and these studies should place increased focus on the need for fusion, instrumentation, and extent of decompression.

## CONCLUSION

Our case suggests that limited decompression may be sufficient for complete resolution of a spinal epidural abscess, thus reducing the morbidity associated with more extensive laminectomies. Although treatment algorithms have been proposed to aid in decision-making, little guidance is provided regarding the extent and manner of surgical intervention. Given the rarity of this condition, much of the literature is composed of case reports and retrospective studies. Further investigation is needed to determine which patients may be suitable for medical management and the type of surgical intervention necessary for symptom and abscess resolution.

## Notes

*This article meets the Accreditation Council for Graduate Medical Education and the American Board of Medical Specialties Maintenance of Certification competencies for Patient Care and Medical Knowledge.*
